# Demonstrating the empirical effect of population specificity of anthropological standards in a contemporary Australian population

**DOI:** 10.1007/s00414-023-03031-z

**Published:** 2023-06-03

**Authors:** Lauren Swift, Zuzana Obertova, Daniel Franklin

**Affiliations:** Centre for Forensic Anthropology, The University of Western Australia, 35 Stirling Hwy, Crawley, WA 6009 Australia

**Keywords:** Sex estimation, Forensic anthropology, Cranial measurements, Population specificity, Sexual dimorphism

## Abstract

The ability to differentiate individuals based on their biological sex is essential for the creation of an accurate anthropological assessment; it is therefore crucial that the standards that facilitate this are likewise accurate. Given the relative paucity of population-specific anthropological standards formulated specifically for application in the contemporary Australian population, forensic anthropological assessments have historically relied on the application of established methods developed using population geographically and/or temporally disparate. The aim of the present paper is, therefore, to assess the accuracy and reliability of established cranial sex estimation methods, developed from geographically distinct populations, as applied to the contemporary Australian population. Comparison between the original stated accuracy and sex bias values (where applicable) and those achieved after application to the Australian population provides insight into the importance of having anthropological standards optimised for application in specific jurisdictions. The sample analysed comprised computed tomographic (CT) cranial scans of 771 (385 female and 386 male) individuals collected from five Australian states/territories. Cranial CT scans were visualised as three-dimensional volume-rendered reconstructions using *OsiriX*®. On each cranium, 76 cranial landmarks were acquired, and 36 linear inter-landmark measurements were calculated using *MorphDB*. A total of 35 predictive models taken from Giles and Elliot (1963), Iscan et al. (1995), Ogawa et al. (2013), Steyn and İşcan (1998) and Kranioti et al. (2008) were tested. Application to the Australian population resulted in an average decrease in accuracy of 21.2%, with an associated sex bias range between − 64.0 and 99.7% (average sex bias value of 29.6%), relative to the original studies. The present investigation has highlighted the inherent inaccuracies of applying models derived from geographically and/or temporally disparate populations. It is, therefore, imperative that statistical models developed from a population consistent with the decedent be used for the estimation of sex in forensic casework.

## Introduction

Methods or standards for the estimation of age, ancestry and stature are often sex specific; accordingly, the accurate and reliable estimation of skeletal sex is a fundamental part of the anthropological assessment [2, 3]. Furthermore, the accurate estimation of sex improves the likelihood of achieving a positive identification by eliminating individuals of the opposite sex from further investigation. Skeletal sex is traditionally estimated based on the morphoscopic and/or morphometric analysis of cranial and post-cranial elements [9, 35], with the most accurate standards attributed to regions exhibiting substantial sexual dimorphism, usually because of divergent evolutionary adaptations (i.e. pelvic shape for childbirth; [21]. The skull is a region of the skeleton that has been shown to exhibit substantial sexual dimorphism to allow for reliable estimations of sex to be achieved [7, 14, 40]. Numerous populations are developing predictive models based on cranial measurements that exceed 80% accuracy, including Japan [22, 27], Greece [23], South Africa [36], Turkey [7] and the USA [15]. The ability to use the skull for estimating sex is based on its preferential preservation during extended periods of exposure [11, 16, 37] and sex-specific morphological variations that are attributed to prolonged skeletal growth of males during puberty and disproportionate musculoskeletal loading between sexes [4].

Of relevance in the accurate estimation of sex is the selection of the most appropriate anthropological method(s). It is also important that the method(s) selected utilise predictive models that are population specific; this concept posits that the most accurate anthropological methods are derived from a population that is geographically and/or temporally consistent with that of the decedent [10]. The underlying biological principle behind population specificity relates to evolutionary adaptations to highly specific environmental stressors that can be extrinsic (geographical or environmental variations) [30, 41] or intrinsic (genetic variations between populations) [17, 32]. These adaptations result in variations in skeletal morphology between discrete populations of individuals. It is, therefore, imperative that highly specific anthropological standards are utilised (where possible) for distinctive populations that most accurately represent their associated morphological expression of sexual dimorphism. To sufficiently recognise the importance of population specificity, it is first appropriate to clearly define the term population. Traditionally, populations were identified as collections of individuals grouped according to a commonality, such as physical appearance [17]. A broadening of the term, however, now groups individuals within a specific geographic location representing a specific country [10]. While this definition is overly simplistic given the transient nature of contemporary humans, it provides a baseline for grouping individuals that is readily and widely understood and facilitates easy comparison.

In Australia, there is a dearth of established cranial sex estimation methods that have been empirically shown to accurately represent the entire population, with only a single study incorporating multiple state/territory samples [38]. Contemporary forensic anthropological research has in turn, focused on sub-populations (e.g. state/territory groupings) [14, 24, 33] with a few examples of collaboration between research institutes across state/territory borders specific to ancestry and stature estimation [2, 19]. Due to the lack of established population-specific anthropological methods, the forensic analysis of unknown skeletal remains within Australia has traditionally relied on utilising established “foreign” predictive models developed for geographically and/or temporally removed populations. While this approach may seem practical, it has not yet been demonstrated whether the application of established predictive models, developed using non-contemporary foreign individuals, facilitates an accurate assessment of sex.

The aim of the present paper is, therefore, to assess the accuracy and reliability of five established cranial sex estimation methods, developed from four geographically and temporally distinct populations, as applied to the contemporary Australian population. The selection of standards is based on geographic proximity to Australia and the original reported accuracy rates. The overall objective is to evaluate the effect of population variance relative to anthropological profiling.

## Materials and methods

### Materials

The sample analysed comprised 771 (385 females and 386 males) computerised tomography (CT) cranial scans, representing adult patients who presented for clinical evaluation in Western Australia, New South Wales, Tasmania, South Australia and the Northern Territory between 2013 and 2019. The sample collectively represents five of the eight Australian states and territories. The overall mean female age was 55.1 years (SD 22.2) with a range of 17.8 to 97.5 years; the mean male age was 48.2 years (SD 21.0) with a range of 17.9 to 96.6 years. Collectively, the sample is intended to be a representation of the contemporary Australian population with respect to age, sex and ancestry.

The use of virtual medical modalities in lieu of physical specimens is well established in the literature and is an acceptable method for analysing morphological variation within living populations [5, 12, 13, 28]. Research ethics approval was granted by the Human Research Ethics Committee of the University of Western Australia (RA/4/1/8926); additional local institutional approval was also granted by the Menzies School of Health Research—Human Research Ethics Committee of the Northern Territory Department of Health (HREC reference number 2017–2879). Prior to collection, scans were anonymised to the investigators with only the date of scan, date of birth (or age when this information was redacted) and biological sex, provided by the medical centres/hospitals; data specific to ancestry is not collected or available (see [12, 13]).

### Methods

#### Visualisation and measurement acquisition

To acquire the requisite measurement data, cranial CT scans were visualised as both two-dimensional radiographic images and three-dimensional (3D) volume-rendered reconstructions using the medical imaging software OsiriX® (3.1.1). A total of 76 landmarks were acquired (definitions as per Swift et al. [38]) in each cranial scan. The x, y and z coordinate data was then used to calculate 36 linear inter-landmark measurements (definitions outlined in Table 1; these calculations were performed using MorphDB (software developed in-house for a database application).

**Table 1:** Definition of the measurements used in the present study; see Swift et al. [38] for landmark definitions

Measurement	Landmarks	Definition
Biasterionic breadth (BAST)	ast-ast	Straight line distance from left to right asterion ^i^
Basion-bregma height (BAB)	b-ba	The distance from basion to bregma ^i^
Basion-nasion (BNL)	ba-n	The distance from nasion to basion ^i^
Prosthion-basion (ABL)	pr-ba	The distance from basion to prosthion ^i^
Maximum cranial length (GOL)	g-op	The straight-line distance from glabella to opisthocranion in the mid-sagittal plane ^i^
Frontal breadth (FRB)	fpt-fpt	Breadth at the coronal suture, perpendicular to the median plane at the temporal line ^v^
Bizygomatic breadth (ZYB)	zy-zy	The maximum breadth across the zygomatic arches, perpendicular to the mid-sagittal plane ^iii^
Foramen magnum length (FML)	fml-fml	The mid-sagittal distance from opisthion to basion ^iii^
Foramen magnum breadth (FMB)	ba–o	Distance between the lateral margins of the foramen magnum at the point of greatest lateral curvature ^iii^
Mastoid height (MHL)	ms-po	The direct distance between porion and mastoidale ^i^
Nasion-Prosthion height (NAH)	n-pr	The distance from nasion to prosthion ^i^
Nasal height (NLH)	n-ins	Average height from nasion to the lowest point on the border of the nasal aperture on either side ^ii^
Nasal breadth (NLB)	al-al	Distance between the anterior edges of the nasal aperture at its widest extent ^i^
Orbit height (OH)	s-or	Height between the upper and lower borders of the orbit. ^i^
Orbit breadth (OB)	zfo-d	Breadth from dacryon to zygofacial approximating the longitudinal axis that bisects the orbit into equal upper and lower parts ^v^
Bimaxillary breadth (MXB)	ifz-ifz	Breadth across the maxilla between zygomaxillare ^i^
Maxillo-alveolar breadth (PAB)	ecm-ecm	The maximum breadth across the alveolar borders of the maxilla measured on the lateral surfaces at the location of ectomalare ^i^
Biorbital breadth (BOB)	fo-fo	Breadth across the face between the most anterior point on the frontomalare suture on either side ^v^
Biauricular breadth (BAE)	ae-ae	The least exterior breadth across the roots of the zygomatic processes ^i^
Interorbital breadth (IOH)	d-d	Distance between left and right dacryon ^i^
Upper facial breadth (BIB)	fmt-fmt	The distance between the right and left frontomalare temporale ^iii^
Nasio-occipital length (NOL)	n-op	Maximum length in the mid-sagittal plane, measured from nasion ^i^
Minimum frontal breadth (MFB)	ft-ft	The distance between the right and left frontotemporal ^iii^
Cheek height (CH)	ifz-or	Distance between the lower border of the orbit measured from orbitale to the inferior lateral zygomatic border ^i^
Palate length (PAL)	aic-p	Distance between the anterior incisive canal and the most inferior point on the inter-palatine suture ^v^
Zygomatic height (ZW)	sz-pz	Height of the zygoma between the superior and inferior zygotemporal junction ^v^
Palate width (PAW)	mt-mt	Widest point on the palate between the most posterior point on the alveolar margin of the maxillar ^v^
Nasomaxillary width (NMW)	nm-nm	Distance between the two junctions of the nasomaxillary sutures ^i^
Canine width (CAW)	ca-ca	Distance between the two canines on the maxilla ^v^
Glenoid width (GEW)	ge–ge	Breadth of the zygoma directly above the glenoid fossa ^i^
Palate foramen width (PFW)	pf-pf	Width of the palate at the point of the palatine foramen ^v^
Canine palate width (CPW)	pca-pca	Width of the palate between the two canines ^v^
Maxillar height (MAH)	pr-ins	Distance between the alveolar margin and the nasal spine ^v^
Nasal length (NL)	r-al	Average distance from rhinion to the inferior border of the nasal spine on both sides ^v^
Bieuryonic breadth (BEU)	eu–eu	The maximum width of the skull perpendicular to the mid-sagittal plane wherever is it located except for the inferior temporal line and the immediate area surrounding the latter ^iii^
Bizygotemporal breadth (BPZ)	pz-pz	Direct length between both zygotemporale ^iv^

Key; (i) Howells (1973) [18], (ii) Bass (2005) [1], (iii) Langley et al. (2016), (iv) Franklin et al. (2005) [8], (v) Swift et al. (2022) [38]

#### Statistical analyses

Previously, a comprehensive statistical analysis of the accuracy and reliability of acquiring the 36 cranial measurements using three-dimensional volume-rendered reconstructed CT scans was conducted by Swift et al. [38]. The results of this investigation indicated that inter-observer error was negligible with all measurements falling within acceptable ranges, relative to the statistical tests performed. The technical error of measurement (TEM) was between 0 and 1.5 mm, the relative technical error of measurement (rTEM) was below 5% and the coefficient of reliability (R) for all measurements was ≥ 0.80 (see Swift et al. [38] for results and discussion of the statistical analyses).

The cranial measurements acquired in the Australian sample were then entered into the following “foreign” discriminant function equations: Giles and Elliot [15], USA,Iscan et al. [22],Ogawa et al. [27], Japanese,Steyn and İşcan [36], South African,and Kranioti et al. [23], Greek (Cretan). Leave-one-out classification data are reported where available. A total of 35 discriminant function equations were applied to the Australian sample, and each was assessed based on overall classification accuracy and the sex bias value (calculated as the difference between the classification accuracy of male assessments relative to female; acceptable range between ± 5% [12, 13]). All statistical analyses were performed using IBM SPSS Statistics 25.

## Results

The performance of each of the established standards, as applied to the Australian sample, is considered individually below.

### US population (African and European Americans): Giles and Elliot [15]

**Fig. 1:**
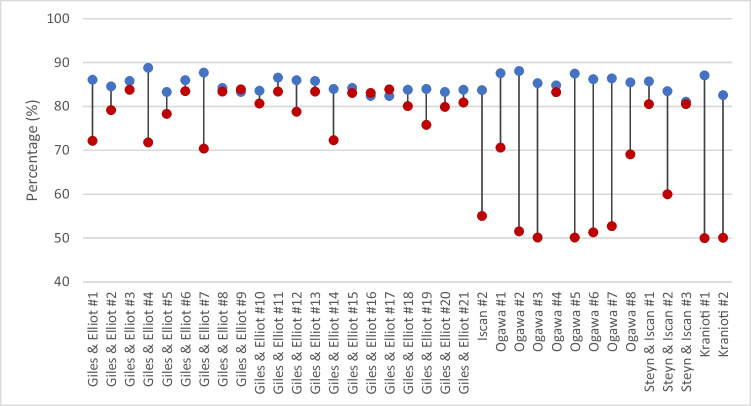
Original published classification accuracies of each applied sex estimation predictive model (blue) compared to the classification accuracies following application to the Australian population (red)

A total of 21 models were applied to the Australian sample, resulting in an average decrease of classification accuracy of 6.1% for 18 of the 21 models (Fig. 1). Functions #7, #4 and #1 were the least accurate when applied to the Australian sample, with respective classification accuracies of 70.4%, 71.8% and 72.2%, and associated sex bias values > 50% (Table 2). These results indicate an extreme misclassification of females in the Australian sample. Interestingly, the accuracy of functions #16 and #17 improved from 82.4% (same original value for both functions) to 83.1% and 83.9%, respectively, when applied to the Australian sample; the associated sex-bias values were also small (− 1.5 and 2.0%, respectively). Unfortunately, the predictive models outlined by Giles and Elliot [15] did not present their original sex bias values, or data sufficient to calculate them,therefore no comparison could be made between the original sex bias values and those achieved following application to the Australian sample.

**Table 2:** Performance of the 21 multivariate discriminant functions by Giles and Elliot (1963) as applied to the Australian population

Discriminant function	Original study	Applied to the Australian population			
	Accuracy (%)	Accuracy (%)			
	Overall	Overall	Female	Male	Sex bias
1	86.1	72.2	46.1	98.2	52.1
2	84.6	79.2	89.3	69.2	− 20.1
3	85.8	83.8	76.6	90.9	14.3
4	88.8	71.8	44.8	98.7	53.9
5	83.3	78.3	94.8	61.9	− 32.9
6	86.0	83.5	74.5	92.5	18.0
7	87.7	70.4	41.9	98.7	56.8
8	84.2	83.4	89.3	77.5	− 11.8
9	83.3	83.9	74.7	93.0	18.3
10	83.6	80.7	70.6	90.7	20.1
11	86.6	83.4	74.0	92.8	18.8
12	86.0	78.8	91.2	66.6	− 24.6
13	85.8	83.4	82.0	84.7	2.7
14	84.0	72.3	47.9	96.6	48.7
15	84.2	83.1	92.2	74.1	− 18.1
16	82.4	83.1	83.9	82.4	− 1.5
17	82.4	83.9	82.8	85.0	2.2
18	83.8	80.1	65.9	94.0	28.1
19	84.0	75.8	55.2	96.4	41.2
20	83.3	79.9	89.1	70.9	− 18.2
21	83.8	80.9	69.8	92.0	22.2

### Japanese population; Iscan et al. [22] and Ogawa et al. [27]

The application of a single predictive model from Iscan et al. [22] to the Australian population resulted in an overall classification accuracy of 55.1%, a decrease of 28.6% from the original published classification accuracy (Fig. 1). The associated sex bias values were likewise significant, decreasing from 1.3% in the original study to − 63.97% (Table 3). These results indicate an extreme misclassification of Australian males.

**Table 3:** Performance of the remaining 14 multivariate discriminant functions as applied to the Australian population. Results are subcategories based on the original study

Discriminant function	Original Studies				Applied to the Australian population			
	Accuracy (%)				Accuracy (%)			
	Overall	Female	Male	Sex Bias	Overall	Female	Male	Sex Bias
Iscan et al. [22] *N* = 76 (32 female; 44 male)*								
2	84.1	82.8	84.1	1.3	55.1	95.3	31.3	− 64.0
Ogawa et al. [27] *N* = 113 (40 female; 73 male)								
1	87.6	88.6	87.1	− 1.5	70.7	46.6	94.6	48.0
2	88.1	88.6	87.2	1.4	51.6	4.5	98.5	94.0
3	85.3	84.6	85.7	1.1	50.1	9.9	97.9	88.0
4	84.8	88.6	82.9	− 5.7	83.3	77.9	88.6	10.7
5	87.5	88.6	87.0	− 1.6	50.1	76.0	84.2	8.2
6	86.2	89.7	84.3	− 5.4	51.3	4.8	97.7	92.9
7	86.4	89.7	84.5	− 5.2	52.7	6.0	99.2	93.2
8	85.5	84.6	85.9	1.3	69.1	20.3	97.7	77.4
Steyn and Iscan (1998) *N* = 91 (47 female; 44 male)								
1	85.7	85.1	86.4	1.3	80.5	64.6	96.4	31.8
2	83.5	80.9	86.4	5.5	60.0	24.2	94.3	70.1
3	81.1	82.6	79.5	− 3.1	80.5	72.0	88.9	16.7
Kranioti et al. [23] *N* = 178 (88 female; 90 male)								
1	87.1	86.9	87.2	0.3	50.0	0.0	99.7	99.7
2	82.6	79.5	85.6	6.1	50.1	0.2	99.7	99.5

^*^Sample size varied according to cranial measurement, reported number of individuals in accordance with the smallest sample used in the equation

**Fig. 2:**
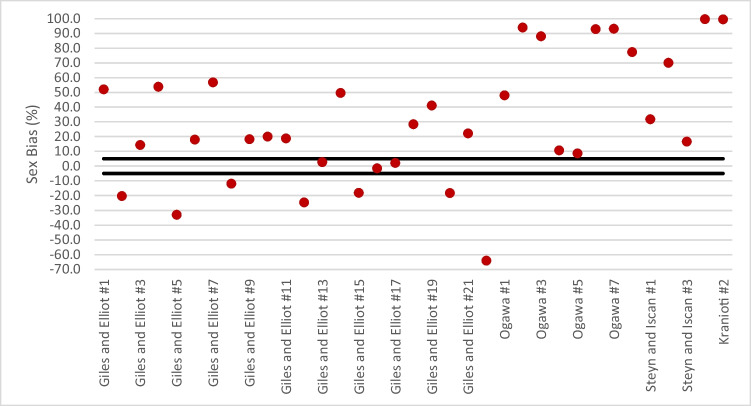
Sex bias values associated with each predictive model following application to the Australian sample. The acceptable range is demarcated by black lines

Similarly, following the application of eight discriminant functions from Ogawa et al. [27], classification decreased relative to the original leave-one-out accuracy by an average of 26.6% when applied to the Australian sample (Fig. 1). Function #5 had the largest discrepancy with a decrease in accuracy from 87.5 to 50.13%. All eight models were also associated with inappropriately large sex bias values, four of which exceeded 80% (Fig. 2), indicating a significant disproportionate misclassification of females in the Australian sample.

### South African population (European ancestry sample): Steyn and Iscan (1998)

A total of three models outlined by Steyn and İşcan [36] were applied to the Australian population, resulting in an average decrease in classification accuracy of 9.8% relative to the original (Fig. 1). The most substantial difference was for function #2, with classification accuracy decreasing from 83.5 to 60.0%. Sex bias values for all three models were unacceptable, ranging between 16.7 and 70.1%, indicating disproportionate misclassification of females (Fig. 2).

### Greek population: Kranioti et al. [23]

Two models from by Kranioti et al. [23] were applied to the Australian population (Fig. 1). The classification accuracy of both was 50%, a decrease of > 30% from the original reported leave-one-out accuracies. The sex bias values were the highest of any of the applied standards at 99.7% and 99.5%, respectively (Fig. 2), indicating a total failure to correctly classify Australian females.

## Discussion

In a forensic context it is of paramount importance that investigators have assigned the correct skeletal sex (i.e. female or male) to a set of unknown remains. This is crucial when it comes to applying sex-specific predictive models for age, stature and ancestry, which are more accurate when sex specific. Thus, the accurate estimation of sex is inherently intertwined with the accuracy of biological analyses and other avenues of investigation performed thereafter.

In the present study, the application of cranial sex estimation methods derived using populations geographically and/or temporally removed from Australia saw a general reduction in classification accuracy (albeit to varying degrees), with nearly all cases having an unacceptably large associated sex bias value. These results indicate that the use of established sex estimation models in an Australian context would be ineffective and highly inaccurate. The reasons underpinning the ineffectiveness of these models relate to overall differences in the cranial morphology between the various populations. A comparison of the mean values of nine common cranial measurements across each of the five populations highlights such variances (Table 4). A common trend is that Australian females are on average larger than females from the USA, Japanese, South African and Greek populations. Assessment of the average measurement values identified that Australian females are more similar in size to the males from those populations. For example, the mean basion-nasion length (BNL) in the female Australian sample is 101.1 mm, which is consistent with the mean male values (101.0 to 102.5 mm) of the other populations.

**Table 4:** Mean values (in mm) of nine common cranial measurements taken on the five distinct populations

Measurement*	Australia	USA	Japan	South Africa	Greece
Female					
GOL	181.0	174.7	171.4	179.0	172.9
BNL	101.1	95.7	97.0	96.2	96.3
BEU	130.6	136.4	139.1	NT	133.9
MFB	97.3	NT	91.3	93.6	93.2
BAB	135.1	127.1	133.5	130.5	132.5
BIB	102.4	NT	95.6	NT	93.2
ZYB	124.9	123.6	128.9	121.9	122.1
FRB	114.0	NT	115.5	113.3	119.0
MHL	24.6	25.8	29	NT	28.6
Male					
GOL	189.0	183.6	179.0	187.7	181.1
BNL	106.8	101.0	102.5	102.4	102.0
BEU	132.8	141.2	144.1	NT	137.6
MFB	99.9	NT	95.3	97.8	96.3
BAB	141.0	133.2	140.4	136.8	139.7
BIB	106.1	NT	100.5	NT	96.3
ZYB	132.0	132.6	135.9	128.9	130.5
FRB	117.0	NT	119.5	119.2	122.5
MHL	28.1	29.2	33.0	NT	31.7

^#^Measurements as per Swift et al. (2022), *NT*, measurement not taken

Similarly, the mean maximum cranial length (GOL) in the female Australian sample is 181.1 mm which is relatively similar and, for some populations, larger than the foreign males (179.0 mm—Japanese and 181.8 mm—Greek). The same pattern was also observed in minimum frontal breadth (MFB) and bifrontal breadth (BIB); see Table 4. Therefore, the application of foreign models using those measurements will result in most females being misclassified as males. This is clearly elucidated when one considers the data shown in Fig. 2: 25 of the 35 models notably misclassified Australian females as male, leading to very large sex bias values.

Morphological variation between the populations is also evident when considering the expression and magnitude of sexually dimorphic features; this can, to some degree, be discerned by examining the relative loadings of the coefficients in the discriminant equations. In examining function #2 of Iscan et al. [22], mastoid height contributed the most to the estimation of sex (i.e. had the highest loading), whereas in Swift et al. [38], which uses the same Australian population as the present study, mastoid height had a proportionately smaller loading and thus contributes less to the model (e.g. relatively lower dimorphism). A similar trend was also observed relative to basion-bregma height for function #2 of Iscan et al. [22], functions #1, #4, #6, #7, #8 and #9 of Giles and Elliot [15] and functions #3, #5 and #8 of Ogawa et al. [27]. For the aforementioned studies, basion-bregma height was ranked within the top three highest loaded (most dimorphic) cranial measurements for each population, contributing significantly toward the estimation of sex. Relative to Swift et al. [38], basion-bregma was not one of the most dimorphic measurements. What this demonstrates is that it is not only a gross size difference, but the variance in which measurements are most strongly weighted (e.g. dimorphic) in the multivariate models. To achieve accurate and reliable results, the latter needs to be optimised to suit the specific population of interest.

The underlying aetiology of cranial morphological variation is related to a combination of factors, including mechanical loading of craniofacial muscles, subsistence patterns [25] and climate [20]. Considering the loading of craniofacial muscles, Schlager and Rudell [34] investigated variation in the zygomatic region of the skull between a Chinese and German population. The investigation demonstrated that 9.7% of the overall variation of the sample was related to population, and further to this, population affinity could be reliably predicted at 97.9% accuracy. The authors hypothesised that the morphological variability of the zygomatic region was directly related to the variations in physical stress caused by mechanical loading of masticatory muscles and differences in the position of insertion points for the *masseter* and *temporalis* muscles. The lateral rim of the orbit was particularly affected, resulting in more pronounced development associated with increased muscle loading in the Chinese population.

The variation in the muscles of mastication is likely an evolutionary difference relative to subsistence, which persists in some contemporary populations. Noback and Harvati [25] investigated the effect that different subsistence methods had on cranial development in 15 discrete *Homo sapiens* populations. Their data suggest that individuals living on diets that comprise tougher harder foods (meat and fish) were associated with more robust, broader skulls, relative to the zygomatic and temporal regions and the alveolar processes. Those populations that survived on more agriculturally based diets, including higher amounts of grain and “processed” food, tended to have a relatively narrower craniofacial region. The theory that populations exhibiting increased loading of the masticatory muscles result in broader zygomatic and temporal regions of the skull, regardless of sex, is supported by several other studies, including Prado et al. [31], von Cramon-Taubadel [39], Noback and Harvati [26] and Paschetta et al. [29].

Climate is another factor that can explain the variations in cranial morphology exhibited between geographically disparate populations. Hubbe et al. [20] analysed craniometric data (33 measurements) in 7422 males from a total of 135 geographic populations, with the aim of exploring the impact of climate on cranial morphology. The results of this investigation indicated a statistically significant correlation between geographic location and cranial morphology, with different anatomical regions of the skull impacted disproportionately. Individuals from colder climates, such as Northern Europe, Northeast Asia and the extreme North of America, characteristically exhibited broader neurocrania. This was interpreted as being a necessary (selectively advantageous) morphological attribute that decreases the surface/volume ratio of the skull and brain, necessary for reducing heat loss through the skull. Individuals from the colder climates were also characterised by morphological changes to the viscerocranium. Those from Northern Europe experienced variations in facial projection, specifically increased nasal and frontal breadth, while those from Northeast Asia and the extreme North of America were characterised by increased nasal height, facial height and breadth. The authors postulated that the most likely cause of these adaptations relates to the need for reduced nasal indexes necessary for warming air during inhalation in cold climates [6].

## Conclusion

The accurate estimation of sex is essential toward providing an accurate biological profile for a set of skeletal remains, with the statistical model applied being crucial to ensuring judicially reliable results. The present investigation has highlighted the inherent inaccuracies of applying models derived from geographically and/or temporally disparate populations. Underlying genetic and epigenetic differences between populations result in variations in cranial morphology and the magnitude of expression of sexually dimorphic features across the skull that are specific to each population. It is, therefore, imperative that statistical models developed from a population consistent with the decedent be used for the estimation of sex in forensic casework.

## Data Availability

The data that support the findings of this study are available from the corresponding author, [DF], upon reasonable request.
